# Diagnostic and surgical challenges of pulmonary mucoepidermoid carcinoma: a case report

**DOI:** 10.1093/jscr/rjag650

**Published:** 2026-07-29

**Authors:** Yeni Arroyave-Guerrero, Luis F Giraldo-Cadavid, Juan Carlos Garzón, Luis Jaime Tellez, Julián A Ariza Traslaviña, Lucia Viola, Nelson Páez-Espinel

**Affiliations:** Universidad El Bosque/National Cancer Institute, 9th Avenue No.131A–02, Bogotá 110111, Colombia; Interventional Pulmonology Service, Fundación Neumológica Colombiana, 13b Avenue No.161-85, Bogotá 111321, Colombia; Department of Thoracic Surgery, Fundación Cardioinfantil, 163A Street No. 13B-60 Bogotá 1113111, Colombia; Department of Thoracic Surgery, Fundación Cardioinfantil, 163A Street No. 13B-60 Bogotá 1113111, Colombia; Department of Thoracic Surgery, Fundación Cardioinfantil, 163A Street No. 13B-60 Bogotá 1113111, Colombia; Thoracic Oncology Unit, Fundación Neumológica Colombiana, 13b Avenue No.161-85, Bogotá 111321, Colombia; Interventional Pulmonology Service, Fundación Neumológica Colombiana, 13b Avenue No.161-85, Bogotá 111321, Colombia

**Keywords:** mucoepidermoid carcinoma, cone-beam CT, airway obstruction, pneumonectomy, multidisciplinary health team, case reports

## Abstract

Pulmonary mucoepidermoid carcinoma (MEC) is a rare salivary gland-type tumour often associated with delayed diagnosis due to nonspecific findings. We report a 75-year-old woman with chronic respiratory symptoms initially treated as asthma without improvement. Computed tomography showed a left hilar lesion with post-obstructive changes and apparent compression of the left main and lower bronchi, suggesting extensive hilar involvement. Bronchoscopic and endobronchial ultrasound-guided biopsies were nondiagnostic despite persistent airway obstruction. Surgical exploration revealed a localized intrabronchial tumour arising from the left lower lobe bronchus. A left lower sleeve lobectomy with preservation of the upper lobe was successfully performed, avoiding pneumonectomy. Histopathology confirmed a 22-mm low-grade MEC with negative margins and no nodal involvement. The postoperative recovery was favorable. This case highlights the diagnostic challenges of pulmonary MEC and the value of parenchymal-sparing surgery in selected patients with central airway tumours.

## Introduction

Mucoepidermoid carcinoma (MEC) accounts for 0.1%–0.2% of all lung cancers and ~5% of bronchial tumours [1–5]. Due to its rarity and nonspecific symptoms, MEC is often misdiagnosed as another cancer type or as asthma [6, 7].

We report a case of endobronchial MEC presenting diagnostic and surgical challenges. This case was reported in accordance with the Surgical CAse REport (SCARE) 2025 criteria [8].

## Case report

A 75-year-old woman, a former smoker with a 10-pack-year history, presented with an 8-year history of dyspnea, productive cough, and wheezing. She was initially diagnosed with late-onset asthma and treated with inhaled therapy, without improvement. She had recurrent pneumonias and previous bronchoscopies showing severe left main bronchial obstruction with non-diagnostic biopsies. She was referred to our center for further evaluation.

Chest computed tomography (CT) showed chronic atelectasis of the left lower lobe (LLL) with bronchiectasis, a hilar lesion compressing the left main bronchus (LMB) and left lower bronchus (LLB), and multiple ground-glass opacities, predominantly in the left upper lobe (LUL) (Fig. 1). Thoracic magnetic resonance imaging demonstrated a 14 × 20 mm perihilar lesion compressing the LMB, suggestive of lymphadenopathy.

**Figure 1:**
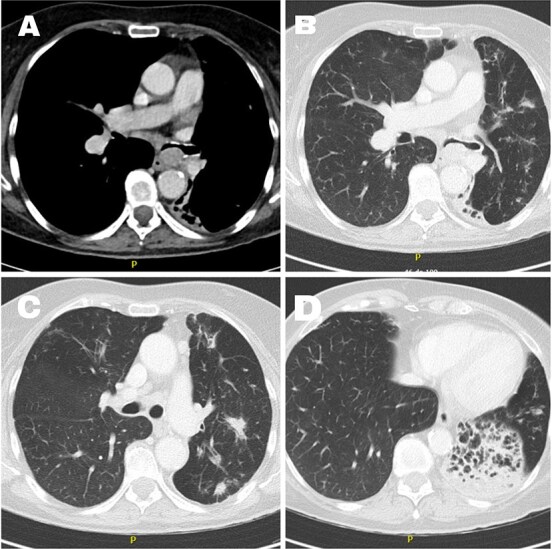
Chest CT: Hilar lesion compressing the LMB. (A) Mediastinal window, (B) lung window, (C) radiopacities in the LUL, (D) chronic atelectasis of the LLL with bronchiectasis.

Bronchoscopy revealed 90% obstruction of the LMB with purulent secretions. A biopsy of the dominant LUL opacity was obtained using cone-beam CT-guided bronchial navigation and radial endobronchial ultrasound (EBUS). Linear EBUS identified a 22 mm heterogeneous LMB lesion and station 7 lymphadenopathy. Biopsies showed inflammatory and reactive changes without malignancy. Microbiological studies and bronchoalveolar lavage cytology were negative. Due to persistent obstruction, bronchial dilation and placement of fully covered self-expanding metallic stent were performed (Fig. 2). However, the stent later migrated and was removed after 7 days.

**Figure 2:**
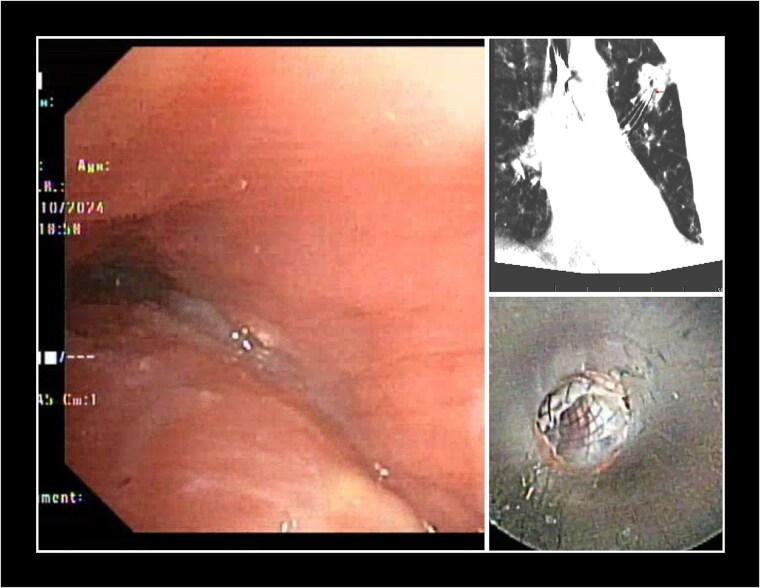
Endoscopic findings. (A) Severe narrowing of the LMB and LLB due to MEC with abundant purulent secretions in the LUL and LLL bronchi. (B) Cone-beam CT with transbronchial biopsy fórceps targeting the center of the LUL lesión. (C) Fully covered self-expanding stent placed via rigid bronchoscopy.

The multidisciplinary team considered the lesion benign but causing significant compression of the LMB and LLB. Given the bronchiectasis, recurrent pneumonias, and persistent symptoms, left lower lobectomy with LMB plasty was recommended, with the possibility of pneumonectomy.

Preoperative evaluation showed preserved cardiac function. Pulmonary function testing demonstrated a forced expiratory volume in 1 second (FEV1) of 1.04 L (57% predicted), forced vital capacity (FVC) of 63% predicted, and adjusted diffusing capacity of the lung for carbon monoxide (DLCO) of 44% predicted. Predicted postoperative FEV1 and DLCO were 45% and 35% after left lower lobectomy, and 30% and 23% after pneumonectomy, respectively. However, ergospirometry demonstrated a peak VO₂ of 12.4 ml/kg/min, and perfusion scintigraphy revealed that the left lung contributed only 13.6% of total pulmonary perfusion. Multidisciplinary assessment considered surgical resection feasible.

Thoracotomy revealed severe pleural adhesions, chronic LLL atelectasis and a hypervascular endobronchial—not hilar—mass occupying 90% of the LMB and involving the left lower bronchus while sparing the LUL bronchus (Fig. 3). A left lower sleeve lobectomy with resection of the bronchial lesion, including the secondary carina and extended proximal and distal margins, was performed. An end-to-end anastomosis between the LMB and the LUL bronchus was completed using PDS 4/0 sutures, followed by mediastinal lymphadenectomy. Intraoperative bronchoscopy confirmed an intact anastomosis.

**Figure 3:**
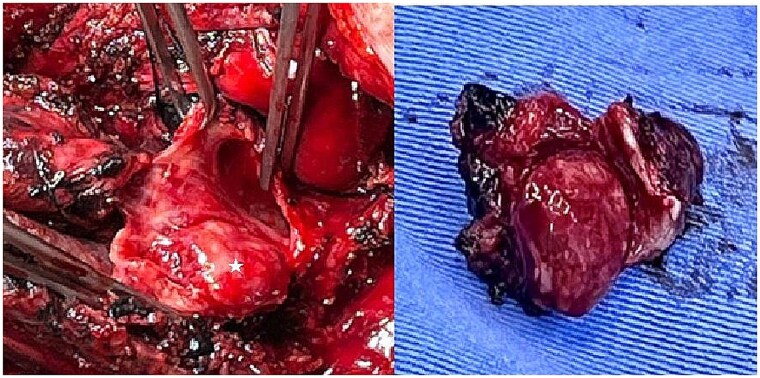
Intraoperative view of the bronchial mucoepidermoid tumour. (A) The LMB has been surgically opened, revealing an endobronchial tumour occupying 90% of the lumen (star). (B) Complete resection of the tumour.

Postoperative recovery was favorable despite a prolonged air leak managed conservatively. Histology revealed a 22 × 17 mm low-grade mucoepidermoid carcinoma without lymphovascular or perineural invasion. Surgical margins and all resected lymph nodes were negative for malignancy. The LLL showed bronchiectasis and extensive fibrosis.

The multidisciplinary team recommended no adjuvant therapy given the low-grade histology, R0 resection, and absence of nodal involvement. At the 18-month follow-up, the patient remained free of respiratory symptoms and recurrent pneumonia. Pulmonary function testing showed an FVC of 73% predicted, an FEV1 of 1.02 L (57% predicted), and an FEV1/FVC ratio of 77%, demonstrating preserved postoperative pulmonary function and improved quality of life.

## Discussion

Primary salivary gland tumors represent <1% of all primary pulmonary tumours and include three subtypes: adenoid cystic carcinoma, MEC, and epithelial-myoepithelial carcinoma [1–4, 6, 7]. MEC is composed of mucin-secreting, squamous, and intermediate cells and is classified as low- or high-grade [1, 2, 9]. Low-grade tumors are usually central and occur in younger patients, whereas high-grade tumors are more aggressive and frequently metastatic [2, 4, 5]. In contrast, our 75-year-old patient presented with an early-stage low-grade central MEC and a prolonged clinical course.

MEC is not strongly associated with smoking or industrial exposures [3] and commonly presents with cough, wheezing, dyspnea, and recurrent pneumonia, although one-third are asymptomatic [1, 3, 9]. Due to its rarity and nonspecific presentation, it is often misdiagnosed as asthma [6, 7, 9]. Our patient was initially treated for asthma without improvement and subsequently developed post-obstructive pneumonia and bronchiectasis with destruction of the LLL.

On CT, MECs typically appear as smooth, central airway masses with homogeneous density and up to 52% of cases show distal obstructive changes such as atelectasis or mucus plugging [1, 4].

Flexible bronchoscopy is the primary diagnostic tool for MEC, typically revealing a smooth, vascular endobronchial mass [1]. However, diagnosis may be challenging with small biopsy samples due to overlapping morphologic features with other pulmonary neoplasms or metastatic salivary gland tumors [7]. Definitive diagnosis may require surgical resection [3].

This case posed significant diagnostic and surgical challenges. Imaging suggested a hilar rather than endobronchial lesion, while severe airway obstruction and purulent secretions limited bronchoscopic assessment and prevented accurate characterization of the obstruction. Multiple biopsies were negative for malignancy. Recurrent post-obstructive pneumonia caused bronchiectasis and severe impairment of pulmonary function, making the patient a high-risk surgical candidate. Nevertheless, advanced functional evaluation and multidisciplinary discussion supported a successful lung-sparing surgical approach.

The mainstay of treatment for MEC is complete surgical resection. Parenchymal-sparing procedures such as sleeve resection may avoid pneumonectomy in centrally located tumors [1–3, 6]. In our case, left lower sleeve lobectomy achieved R0 resection while preserving the LUL.

Adjuvant therapy is generally reserved for advanced disease, incomplete resections, or high-grade tumors [2, 3, 6]. Prognosis depends on tumour grade, TNM stage, and R0 resection status, the 5-year overall survival reaches up to 100% in stage I-II disease but decreases to 43.6% in advanced stages [3, 9]. In this case, the patient had a low-grade, stage IB (T2aN0M0) according to TNM9, with R0 resection, so adjuvant therapy was unnecessary.

In conclusion, pulmonary mucoepidermoid carcinoma may mimic common airway diseases and remain undiagnosed despite negative biopsies. Parenchyma-sparing surgery may provide curative treatment while preserving lung function in selected patients. Management is best performed at multidisciplinary centers with expertise in complex airway surgery.
